# Enrichment of GABARAP Relative to LC3 in the Axonal Initial Segments of Neurons

**DOI:** 10.1371/journal.pone.0063568

**Published:** 2013-05-09

**Authors:** Masato Koike, Isei Tanida, Tomohisa Nanao, Norihiro Tada, Jun-ichi Iwata, Takashi Ueno, Eiki Kominami, Yasuo Uchiyama

**Affiliations:** 1 Department of Cell Biology and Neuroscience, Juntendo University School of Medicine, Tokyo, Japan; 2 Department of Biochemistry, Juntendo University School of Medicine, Tokyo, Japan; 3 Department of Biochemistry and Cell Biology, National Institute of Infectious Diseases, Toyama, Shinjyuku, Tokyo, Japan; 4 Division of Genome Research, Research Institute for Diseases of Old Ages, Juntendo University School of Medicine, Tokyo, Japan; UCL School of Pharmacy, United Kingdom

## Abstract

GABA_A_ receptor-associated protein (GABARAP) was initially identified as a protein that interacts with GABA_A_ receptor. Although LC3 (microtubule-associated protein 1 light chain 3), a GABARAP homolog, has been localized in the dendrites and cell bodies of neurons under normal conditions, the subcellular distribution of GABARAP in neurons remains unclear. Subcellular fractionation indicated that endogenous GABARAP was localized to the microsome-enriched and synaptic vesicle-enriched fractions of mouse brain as GABARAP-I, an unlipidated form. To investigate the distribution of GABARAP in neurons, we generated GFP-GABARAP transgenic mice. Immunohistochemistry in these transgenic mice showed that positive signals for GFP-GABARAP were widely distributed in neurons in various brain regions, including the hippocampus and cerebellum. Interestingly, intense diffuse and/or fibrillary expression of GFP-GABARAP was detected along the axonal initial segments (AIS) of hippocampal pyramidal neurons and cerebellar Purkinje cells, in addition to the cell bodies and dendrites of these neurons. In contrast, only slight amounts of LC3 were detected along the AIS of these neurons, while diffuse and/or fibrillary staining for LC3 was mainly detected in their cell bodies and dendrites. These results indicated that, compared with LC3, GABARAP is enriched in the AIS, in addition to the cell bodies and dendrites, of these hippocampal pyramidal neurons and cerebellar Purkinje cells.

## Introduction

GABA_A_ receptor-associated protein (GABARAP) was first isolated as a protein interacting with the intercellular loop of the gamma2 subunit of GABA_A_ receptor [1], [2], with the human, mouse, rat and bovine orthologs of GABARAP being 100% identical at the amino acid level [2]. GABARAP has been shown to interact with microtubules, tubulin, N-ethylmaleimide (NEM)-sensitive factor, gephyrin, Unc-51-like kinases, Nix, PRIPs (phospholipase C-related catalytically inactive proteins), and NaPi-IIa (Na^+^-dependent Pi-cotransporter IIa) [1], [3], [4], [5], [6], [7], [8], [9] and to co-localize with NEM-sensitive factor to the intercellular membrane compartments and subsynaptic cisternae of cultured neurons. PRIP-1 regulates GABA_A_ receptor surface expression by inhibiting the interaction between GABARAP and GABA_A_ receptor. Disruption of the PRIP-1 gene in mice results in the impairment of Zn^2+^ modulation of GABA-induced Cl^−^ current in hippocampal neurons, the inhibition of motor coordination and alterations in GABA_A_ receptor pharmacology. Although studies have focused on the interaction of GABARAP with GABA_A_ receptor in neurons, GABARAP expression is ubiquitous, being observed in the rat liver, testes, lungs, spleen, thyroid gland, heart, skeletal muscle, and kidneys [10]. Interestingly, GABARAP deficiency has been shown to modulate the expression of NaPi-IIa in renal brush borders [9].

GABARAP has also been characterized as a mammalian homolog of the yeast Atg8 (autophagy-related protein8)/Apg8/Aut7; other homologues include LC3 (microtubule-associated protein 1 light chain 3), Golgi-associated ATPase enhancer of 16 kDa (GATE-16)/GABARAPL2/GEF2, and Atg8L [10], [11]. GABARAP is also highly homologous with the other Atg8 homologs, especially with Atg8L/GEC1/GABARAPL1 (87% identity and 97% similarity) and GATE-16 (57% identity and 97% similarity) [4], [12], [13], [14], [15], [16]. We have shown that GABARAP, along with the other Atg8 homologs, can act as a ubiquitin-like modifier protein, and that GABARAP modification is mediated by the cysteine protease Atg4B, the E1-like enzyme Atg7/Apg7, and the E2-like enzyme Atg3/Apg3 [10], [11], [17], [18], [19], [20]. Although LC3 lipidation has been shown to occur during autophagy in many cell lines and tissues, little endogenous GABARAP lipidation occurs during autophagy in rat heart, liver, kidney, and brain [21]. Rather, GABARAP lipidation has been observed only during the differentiation of C2C12 cells to myotubes independent of the mTor-signaling pathway, suggesting that GABARAP and its lipidation are involved in cellular functions other than autophagy. Using recently generated GFP-GABARAP transgenic mice, we found that the GFP-GABARAP/p62 complex is responsible for the impairment of glomerular function under certain pathological conditions [22].

Despite the importance of GABARAP to neuron function, the distribution of this protein in neurons remains unclear. Using GFP-GABARAP transgenic mice, we investigated the intracellular distribution of GFP-GABARAP in hippocampal pyramidal neurons and cerebellar Purkinje cells by comparing its distribution with that of endogenous LC3. We found that GABARAP and LC3 show different distribution patterns in these neurons.

## Results

### Little of the GFP-GABARAP in GFP-GABARAP transgenic mice is sensitive to starvation

**Figure 1:**
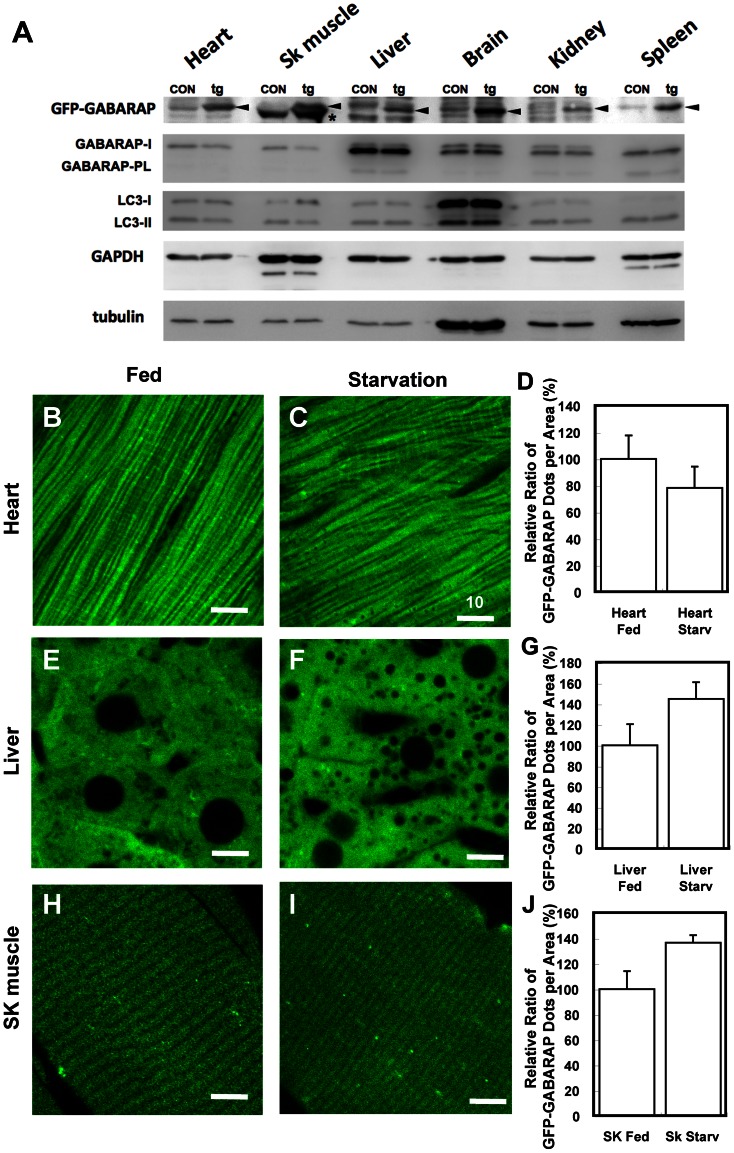
Starvation conditions have little effect on GFP-GABARAP expression in the heart, liver and skeletal muscle. (A) Expression of GFP-GABARAP in tissues of GFP-GABARAP transgenic mice. Cell lysates were immunoblotted with anti-GFP antibody to detect GFP-GABARAP. Endogenous GABARAP and LC3 were detected with anti-GABARAP and anti-LC3 antibodies, respectively. Arrow-heads indicate GFP-GABARAP; asterisks indicate non-specific bands in the skeletal muscle. CON, lysate from control mouse tissue; tg, lysate from GFP-GABARAP transgenic mouse tissue; SK muscle, skeletal muscle. (**B**–**I**) Confocal fluorescence images of GFP-GABARAP in the **heart** (**B** and **C**), **liver** (**E** and **F**), and **SK muscle** (**H** and **I**). Fluorescence of GFP-GABARAP was observed under confocal laser-scanning microscopy (FV1000: Olympus), and GFP-GABARAP dots were counted using an ImageJ program (http://rsbweb.nih.gov/ij/) with a TopHat plugin (http://rsb.info.nih.gov/ij/plugins/lipschitz/). (**D**, **G**, and **J**) Relative ratios of GFP-GABARAP dots per unit area in the heart (**D**), liver (**E**), and skeletal muscle (**J**), using at least 10 images from each tissue in four mice. **Fed**, tissues under fed conditions; **Starvation**, tissues under starvation conditions for 48 h. Bars indicate 10 mm.

GABARAP is highly homologous to the autophagosomal marker LC3. In contrast to LC3, however, little endogenous GABARAP is lipidated in mouse tissues, including the heart, liver, and kidneys, even during autophagy, whereas LC3 is lipidated to form LC3-II (LC3-phospholipid conjugate) [21]. To investigate the distribution of GABARAP in neurons, we generated GFP-GABARAP transgenic mice. GFP-GABARAP was expressed in almost all tissues examined (Figure 1A), although its expression had little effect on the levels of endogenous GABARAP and GABARAP-lipidation in these mouse tissues.

We next investigated whether GFP-GABARAP puncta were increased under starvation conditions in the heart, liver and skeletal muscle of these GFP-GABARAP transgenic mice. In GFP-LC3 transgenic mice, GFP-LC3 puncta in these organs increased under starvation conditions because of LC3-lipidation [23]. We have reported that little endogenous GABARAP is lipidated in mouse tissues under starvation conditions [21]. If, in GFP-GABARAP transgenic mice, this protein is lipidated under starvation conditions, then increased GFP-GABARAP puncta would be observed in their hearts, livers and skeletal muscles; if not lipidated, there will be little increase in these puncta. We observed few GFP-GABARAP puncta in the heart (Figure 1B–D), and similar numbers in the liver and skeletal muscles (Figure 1E–J) under fed and starvation conditions. These findings indicated that little GFP-GABARAP was sensitive to starvation-induced autophagy, similar to our previous findings on the lipidation of endogenous GABARAP [21], [24].

### Most endogenous GABARAP in the brain is GABARAP-I, an unlipidated form

**Figure 2:**
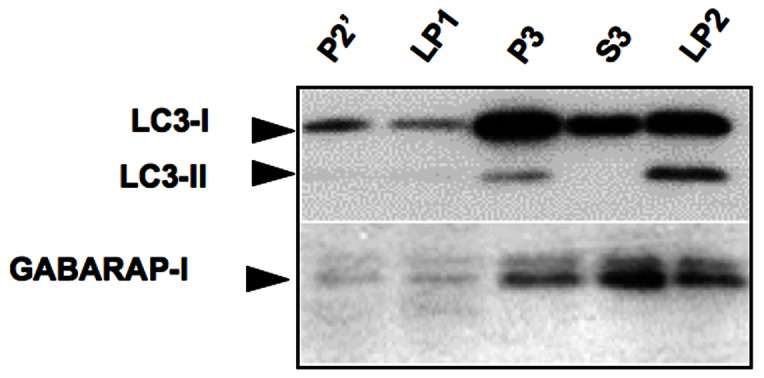
Most endogenous GABARAP in the brain is GABARAP-I. A crude synaptosomal fraction (P2') was prepared from mouse brain, and the residual supernatant containing small cell fragments such as microsomes and soluble proteins was centrifuged at 100,000×g to yield P3 pellet (microsome-enriched fraction) and S2 supernatant (soluble protein-enriched fraction). After hypotonic lysis of the P2' fraction, the lysate was centrifuged at 33,000×g to yield the lysate-pellet (LP1) and the lysate-supernatant (LS1). LS1 was further centrifuged at 260,000×g for 2 h. After discarding the supernatant, the pellet (LP2) was collected as a synaptic vesicle-enriched fraction. Note that little GABARAP-PL was present in any fraction, while LC3-II was present in both the microsome-enriched and synaptic vesicle-enriched fractions.

GABARAP has been shown to localize to intercellular membrane compartments and subsynaptic cisternae in cultured neurons [25]. We performed subcellular fractionation of mouse brain, and investigated whether endogenous GABARAP in the fractions containing membranous compartments is GABARAP-I or GABARAP-phospholipid conjugate (GABARAP-PL). Crude synaptosomal, microsome-enriched, and soluble protein-enriched fractions were prepared from mouse brain homogenates (Figure 2, P2', P3, and S3 respectively), and a synaptic vesicle-enriched fraction was prepared from a crude synaptosomal fraction by hypotonic lysis and differential centrifugation (Figure 2, LP2). GABARAP-I was present in each fraction, especially in microsome-enriched, soluble protein-enriched, and synaptic vesicle-enriched fractions. Little GABARAP-PL was present in any fraction. In contrast, LC3-II was present in the microsome-enriched and synaptic vesicle-enriched fractions. The results indicated that, in the brain, most GABARAP is present in its unlipidated form, GABARAP-I, and suggested that GABARAP localizes to membranous compartments, independent of its lipidation.

### GFP-GABARAP preferentially localizes to the axonal initial segments of neurons, in addition to dendrites and cell bodies

**Figure 3:**
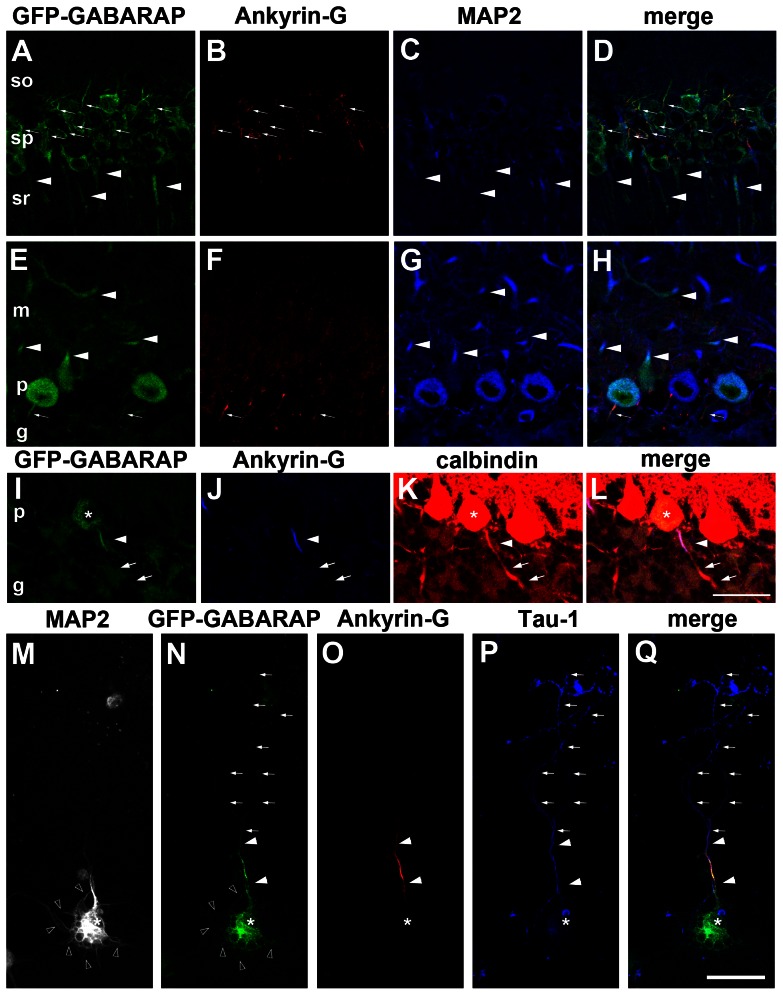
GFP-GABARAP colocalizes with ankyrin-G and MAP2. (A–H) The pyramidal neurons of the CA1 region of the hippocampus (A–D) and cerebellar Purkinje cells (E–H) of GFP-GABARAP transgenic mice were stained with antibodies to GFP (green), ankyrin-G (red) and MAP2 (blue). GFP and MAP2 co-localized in the dendrites of hippocampal pyramidal and cerebellar Purkinje cells in the stratum radiatum (sr) and molecular layer (m), respectively (arrowheads). GFP was also detected in the ankyrin-G-positive axonal initial segments of these neurons (arrows). (I–L) Calbindin-positive axons of Purkinje cells of GFP-GABARAP transgenic mice (an asterisk), specifically axonal segments positive for ankyrin-G (blue) and calbindin (red) (arrow), were strongly positive for GFP signal (green), whereas distal axons devoid of ankyrin-G immunoreactivity (arrows) were not. (M–Q) Cultured cortical neurons from GFP-GABARAP transgenic mice immunostained for MAP2 (white), GFP (green), ankyrin-G (red) and tau-1 (blue). The asterisk indicates the cell body of a GFP-GABARAP-positive neuron. Weak immunoreactivity for GFP was detected in MAP2-positive dendrites (open arrowheads) (M, N). (N–Q) Strong GFP-GABARAP signals detected in the cell body and axon initial segment, which were positive for ankyrin-G and tau-1. In contrast, little GFP signal could be detected in tau-1-positive distal axons devoid of ankyrin-G immunoreactivity (arrows). Abbreviations: so, stratum oriens; sp stratum pyramidale; p, Purkinje cell layer; g, granular cell layer. Bars indicate 30 µm.

**Figure 4:**
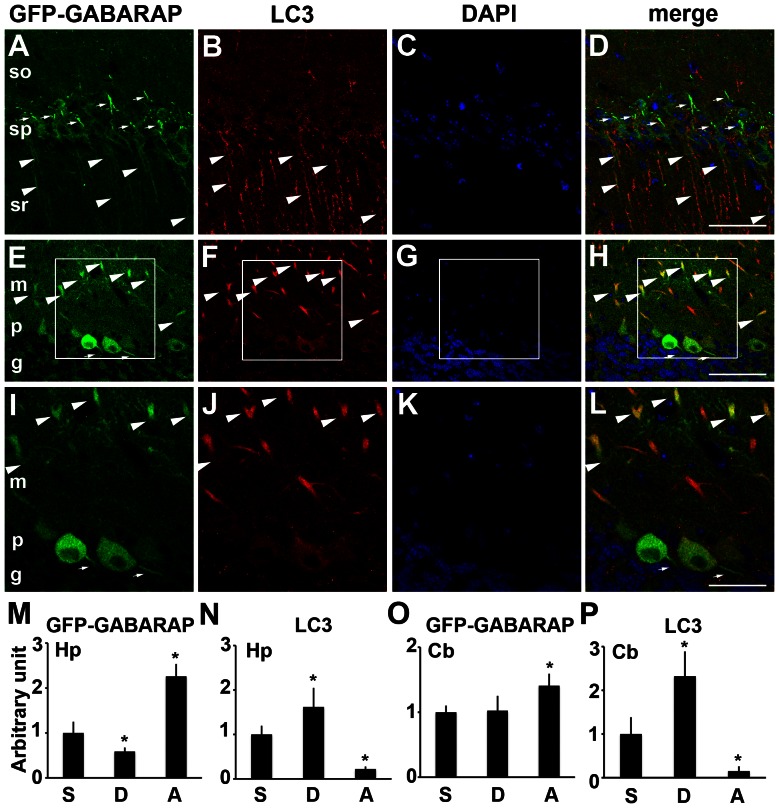
GFP-GABARAP, but little LC3, is enriched in the axonal initial segments of neurons. (A–L) Distribution patterns of GFP-GABARAP (green) and endogenous LC3 (red) in the pyramidal neurons of the CA1 region of the hippocampus (A–D) and cerebellar Purkinje cells (E–L) of GFP-GABARAP transgenic mice. Boxed areas in E–H are enlarged and shown in I-L. Nuclei were stained with DAPI (blue). Immunopositive signals for GFP and LC3 co-localized in the dendrites of hippocampal pyramidal and cerebellar Purkinje cells located in the stratum radiatum (sr) and the molecular layer (m), respectively (arrowheads). Intense immunopositivity for GFP, but not for LC3, was present along the axonal initial segments of these neurons (arrows). (M–P) Fluorescence intensity of GFP (M, O) and LC3 (N, P) immunoreactivity in the somata (S), dendrites (D) and axon initial segments (A) of pyramidal neurons in the hippocampus (Hp) (M, N) and Purkinje cells in the cerebellum (Cb) (O, P). Intensities are normalized relative to those of somata. Vertical bars represent means ± SEMs (n = 10 and 7 for hippocampal neurons and cerebellar Purkinje cells, respectively). Both in the hippocampal neurons and cerebellar Purkinje cells, the intensity of GFP immunoreactivity was highest in the axonal initial segments, whereas the highest immunoreactivity for LC3 was detected in the dendrites (*P<0.01, one-way ANOVA followed by Tukey's *post hoc* test). The intensity of LC3 immunoreactivity in the axon initial segments was almost negligible. Abbreviations: so, stratum oriens; sp, stratum pyramidale; p, Purkinje cell layer; g, granular cell layer. Bars indicate 30 µm in (A–H) and 60 µm in (I–L).

Since GABARAP is a key regulator of GABA_A_ receptor function [2], we first tried to examine the distribution of endogenous GABARAP in the hippocampus and cerebellum using our antibodies against GABARAP raised against GST-fused human GABARAP or synthetic peptides corresponding to residues 8–22 of human GABARAP [21], [22]. However, we found that the antibody did not work for immunohistochemical analyses (Figure S1). Therefore, we investigated the distribution of GFP-GABARAP in the hippocampal and cerebellar neurons of GFP-GABARAP transgenic mice. We observed widespread expression of GFP-GABARAP in the neurons of various regions of the brain, including the cerebral cortex (data not shown), the hippocampus and the cerebellum (Figure 3 & 4). Interestingly, intense immunopositivity for GFP-GABARAP was detected along the axonal initial segments (AIS) of hippocampal pyramidal neurons and cerebellar Purkinje cells, with diffuse and/or fibrillary staining patterns observed in the cell bodies and dendrites of these neurons. Triple staining for GFP, the dendrite marker MAP2 and the AIS marker ankyrin-G [26] confirmed that GFP-GABARAP is enriched in the AIS, as well as cell bodies and dendrites (Figure 3). We also confirmed co-localization of signals for GFP and pan voltage gated sodium channel, which is densely accumulated in the AIS [27], although treatment with antigen retrieval solution diminished the GFP signal to some extent (Figure S2).

Our finding of endogenous GABARAP in the synaptosomal fraction (Figure 2) suggested that GABARAP is present in axons. To confirm whether the intense signal for GFP-GABARAP is observed within AIS, we performed triple stained cerebellar Purkinje cells for GFP, ankyrin-G and calbindin (Figure 3I–L). By overexposing immunofluorescent signal for calbindin, we visualized Purkinje cell axons crossing in the granular layer (Figure 3K). Triple staining showed a strong GFP signal within the AIS, as shown by staining for ankyrin-G and calbindin. Our immunohistochemical analyses showed that the GFP signal was hardly detectable in distal axons unreactive with antibody to ankyrin-G.

To further confirm whether GFP-GABARAP is enriched within AIS, we prepared cultured cortical neurons from GFP-GABARAP transgenic mice, and incubated these neurons with antibodies to MAP2, GFP, ankyrin-G and tau-1 (Figure 3M–Q). Weak immunoreactivity for GFP was detected in MAP2-positive dendrites (Figure 3M, N). Besides the cell body, intense signal for GFP-GABARAP was detected in AIS doubly positive for ankyrin-G and tau-1. In contrast, GFP was hardly detectable in tau-1-positive distal axons negative for ankyrin-G (Figure 3N–Q). These results strongly suggested that GFP-GABARAP is enriched within the AIS of the neurons, both *in vitro* and *in vivo*.

### GFP-GABARAP is enriched in the axonal initial segments in addition to dendrites and somata, while LC3 is not

Although LC3 has been shown to localize to dendrites and cell bodies, endogenous LC3 has never been reported to localize along the AIS of the hippocampal and cerebellar Purkinje neurons [28], [29]. In GFP-LC3 transgenic mice, GFP-LC3 is intensely localized in dendrites and cell bodies but not enriched in AIS [30]. GABARAP has been reported to colocalize with LC3 in nutrient-starved cells and in differentiated C2C12 myotubes [11], [21]. To determine whether GFP-GABARAP colocalizes with endogenous LC3 in the AIS of hippocampal pyramidal neurons and cerebellar Purkinje cells, we compared the localization of GFP-GABARAP and endogenous LC3 in these neurons (Figure 4B). As reported previously [28], diffuse and/or fibrillary staining of LC3 was detectable primarily in the dendrites of hippocampal pyramidal neurons and cerebellar Purkinje cells in the stratum lucidum and molecular layer, respectively (Figure 4B, F, J). We also observed diffuse staining of LC3 in the cell bodies of these neurons. Diffuse and/or fibrillary staining of GFP-GABARAP, colocalizing with endogenous LC3, was also observed in the cell bodies and dendrites of these neurons. Along the AIS of these neurons, however, we observed intense staining for GFP-GABARAP but much weaker expression of LC3 (Figure 4A, D, E, H, I, K). By measuring GFP and LC3 fluorescence intensity in the somata, dendrites and AISs of pyramidal neurons in the hippocampus (Figure 4M, N) and Purkinje cells in the cerebellum (Figure 4O, P), we found that, in both, GFP immunoreactivity was highest in the AISs whereas LC3 immunoreactivity was highest in the dendrites and almost negligible in the AISs. These results indicated that, relative to LC3, GFP-GABARAP is selectively enriched in the AIS.

## Discussion

By double staining for GFP and Ankyrin-G or pan voltage gated sodium channels, we have shown here that GFP-GABARAP is enriched in the AIS, in addition to dendrites and somata, of hippocampal pyramidal neurons and cerebellar Purkinje cells, while endogenous LC3 is mainly present in the cell bodies and dendrites of these neurons. This tendency was also observed in *in vitro* cultured neurons prepared from GFP-GABARAP transgenic mice. Even in membranous fractions, GABARAP was present as unlipidated GABARAP-I, suggesting that GFP-GABARAP in the AIS is also unlipidated. Previous studies in the neurons of GFP-LC3 transgenic mice have suggested that, like endogenous LC3, GFP-LC3 is expressed primarily as diffuse and/or fibrillary signals in the cell bodies and dendrites of neurons [28], [31], but is not concentrated in the AIS [30]. These findings indicate that the preferential localization of GFP-GABARAP in the AIS is not due to side effects of its overexpression.

Although GABARAP was found to co-localize with LC3 in nutrient-starved cells and differentiated C2C12 myotubes [11], [21], and we were unable to detect endogenous GABARAP in brain tissues immunohistochemically, our results provide the first direct evidence that GFP-GABARAP and LC3 are differently distributed in neurons. Our immunoblotting and immunohistochemical analyses indicated that both LC3 and GFP-GABARAP are present in neuronal cell bodies, dendrites and axon terminals, whereas GFP-GABARAP, but not LC3, is preferentially enriched in the AIS of the neurons. We also demonstrated, that apart from LC3, most endogenous GABARAP in mouse brain is unlipidated, indicating that the localization of GFP-GABARAP to the AIS may be due to neuron-specific and autophagy-independent functions of the brain. The AIS, a specialized membrane domain located in proximal axons, has been shown to consist of densely clustered voltage-gated sodium channels that generate action potentials; voltage-gated potassium channels that modulate the amplitude, duration, and frequency of these action potentials; cytoskeletal adaptor proteins including ankyrin-G and cell adhesion molecules [32]. Neuronal populations differ in their composition of ion channels and synaptic innervation in the AIS [33], [34]. For example, the alpha2 subunit of the GABA_A_ receptor is enriched along the AIS of hippocampal pyramidal neurons to receive axo-axonic input [35], whereas the AIS of cerebellar Purkinje cells is associated with ramified axons of GABAergic basket cells called the pinceau formation. Although the latter structure is unique, recent precise morphological and immunohistochemical analyses have shown that typical synaptic contact at the AIS of cerebellar Purkinje cells is very rare. Moreover, the alpha1 subunit of the GABA_A_ receptor, a subunit in Purkinje cells, did not form detectable clusters along the AIS, indicating that Purkinje cells do not receive GABA-mediated synaptic inhibition from basket cells on the AIS [36].

GABARAP has been shown to interact with the intercellular loop of the gamma2 subunit of the GABA_A_ receptor and gephyrin [1], [3], [37]. GABARAP regulates the surface expression of GABA_A_ receptor cooperating with PRIP-1, interacts with NSF, and contributes to the secretory pathway [2], [6]. Our finding, that GABARAP is enriched in the AIS of hippocampal pyramidal neurons and cerebellar Purkinje cells, indicates that the function of GABARAP in the AIS may not be associated with the regulation of GABA_A_ receptors. Indeed, the synaptic localization of GABA_A_ receptors was not altered in GABARAP-deficient mice [38]. In addition to binding to the gamma2 subunit of GABA_A_ receptor, GABARAP can bind to many types of membrane proteins, including transferrin receptor, NaPi-IIa, angiotensin II Type 1 receptor, transient receptor potential vanilloid 1, and κ opioid receptor [1], [3], [4], [5], [8], [9], [39], [40], [41]. GABARAP may therefore regulate the trafficking of densely accumulated receptors in the AIS.

What would be the functional implications for AIS enrichment of a particular protein like GABARAP? Analyses of delepetion of GABARAP-interacting proteins in mice suggested potential functions of GABARAP in the AIS. KIF5A regulated neuronal surface expression of GABA_A_ receptors *via* an interaction with GABARAP [42]. KIF5A deletion causes epilepsy [42]. KIF5 has a preference to the microtubules in the AIS [43]. The modulation of GABA-induced Cl^−^ current by Zn^2+^ or diazepam is impaired in hippocampal neurons of PRIP-1 knockout mice [6]. Motor coordination was impaired and the intraperitoneal injection of diazepam induced markedly reduced sedative and antianxiety effects in the mutant mice. PRIP is implicated in the trafficking of gamma2 subunit-containing GABA_A_ receptors to the cell surface, probably by acting as a bridging molecule between GABARAP and the receptors. Therefore, GABARAP may affects KIF5-dependent GABA-mediated ion channels including Cl^−^ channels in the AIS. Further studies for the function of GABARAP in the AIS will be required using the GFP-GABARAP transgenic mice.

## Materials and Methods

### Ethics Statement

The procedures involving animal care and sample preparation were approved by the Animal Experimental Committee of Juntendo University Graduate School of Medicine (Permit number: 240083) and performed in compliance with the regulations and guidelines for the care and use of laboratory animals of Juntendo University Graduate School of Medicine.

### Primary Antibodies

Anti-GABARAP antibodies raised against GST-fused human GABARAP and synthetic peptides corresponding to residues 8–22 (EHPFEKRRSEGEKIR) of human GABARAP have been described [21], [22]. Polyclonal anti-LC3 antibodies were prepared as described [28], [44] or purchased from Cell Signaling Technology, Inc. (#4108). Polyclonal anti-GFP antibody was purchased from Rockland (600-101-215). Goat and guinea pig anti-GFP, goat anti-MAP2, guinea pig anti-calbindin, and rabbit anti-ankyrin-G antibodies were purchased from Frontier Science (Ishikari, Japan) (Af1480, Af1180, Af860, Af280, and Af610, respectively). Rabbit anti-ankyrin-G antibody was purchased from Santa Cruz Biotechnology, Inc. (H-215). Mouse monoclonal antibodies against pan voltage gated sodium channels (clone K58/35) and anti tau-1 (clone PC1C6) were purchased from Sigma-Aldrich (S8809) and Millipore (MAB3420), respectively.

### Preparation of synaptosomal and synaptic vesicle-enriched fractions

Synaptosomal and synaptic vesicle-enriched fractions were prepared from mouse brain as described [45]. Briefly, mouse brain was homogenized in 10 volumes of ice-cold homogenization buffer (320 mM sucrose, 4 mM HEPES-NaOH, pH 7.3) containing 1 µg/ml pepstatin A (Peptide Inst., 4397), 1 µg/ml leupeptin (Peptide Inst., 4041), and 0.2 mM phenylmethylsulfonyl fluoride (Sigma-Aldrich, P7626), using a loose-fitting glass Teflon homogenizer (nine strokes, 900 rpm). The homogenate was centrifuged at 1,000×g at 4°C for 10 min to remove large cell fragments and nuclei, and the resulting supernatant (S1) was centrifuged for 15 min at 12,000×g at 4°C to yield supernatant S2, containing small cell fragments such as microsomes and soluble proteins, and pellet P2. S2 was centrifuged at 100,000×g at 4°C for 2 h to yield pellet P3, a microsome-enriched fraction, and supernatant S3, a soluble protein-enriched fraction. P2 was washed by resuspending in ice-cold homogenization buffer and recentrifuging at 12,000×g at 4°C. The resulting pellet (P2') represented a crude synaptosomal fraction. To release synaptic vesicles from the synaptosomes, P2' was resuspended in homogenization buffer, and transferred into a glass-Teflon homogenizer. After adding 9 volumes of ice-cold water, the fraction was homogenized; 1 M HEPES-NaOH, pH 7.4, containing 1 µg/ml pepstatin A, 1 µg/ml leupeptin, and 0.2 mM phenylmethylsulfonyl fluoride was added; and the resulting suspension was centrifuged at 33,000×g for 20 min to yield the lysate-pellet (LP1) and lysate-supernatant (LS1). LS1 was centrifuged at 260,000×g for 2 h. After discarding the supernatant (LS2), the pellet (LP2) was collected as a synaptic vesicle-enriched fraction.

### Immunoblotting

Mouse tissues were homogenized in 10 volumes of ice-cold 0.25 M sucrose, 5 mM Tris-HCl, pH 7.5, containing Complete® protease-inhibitor cocktail (Roche Diagnostics, 1697498), using a glass Teflon homogenizer. The homogenates were centrifuged at 500×g at 4°C for 10 min to remove debris. Protein concentrations were determined using the BCA protein assay (Thermo Scientific, 23225). Total proteins were separated by SDS-PAGE, and immunoblotting was performed according to standard chemiluminescent method, using SuperSignal West Dura Extended Duration Substrate or SuperSignal West Pico Chemiluminescent Substrate (Thermo Scientific, 34075 and 34077).

### Plasmid construction

The restriction enzymes, *Nhe*I, *Sal*I, and *Xho*I, were purchased from New England Biolabs; R0131, R0138, and R0146 respectively). A 1.1-kb *Nhe*I-*Sal*I DNA fragment containing the human GABARAP cDNA fused to EGFP at the N-terminus (GFP-GABARAP) was excised from pGFP-hGABARAP [17], blunted with *KOD* DNA polymerase (TOYOBO, KOD-101), and inserted into the blunt-ended *Xho*I site of pCAGGS downstream of the cytomegalovirus immediate-early (CMVie) enhancer and chicken-actin (CAG) promoter [46] to generate pCAG-GFP-GABARAP.

### GFP-GABARAP transgenic mice

Generation and genotyping of GFP-GABARAP transgenic mice have been described [22]. Briefly, the 3.4-kb SalI-PstI fragment was isolated from pCAG-GFP-GABARAP and microinjected into the pronuclei of fertilized 1-cell eggs from B6D2F1/Crj mice (C57BL/6NCrj×DBA/2NCrj). Then, 24 microinjected eggs were transferred into the oviducts of pseudopregnant ICR mice. After extraction of DNA from tail biopsies, 20 mice were screened by PCR analysis for incorporation of the transgene, using the primers CAGGS-F (5′-GGCTTCTGGCGTGTGACC-3′) and CAGGS-Rv (5′-AGCCACCACCTTCTGATAG-3′). Another primer set (EGFP-Fw: 5′-ATGGTGAGCAAGGGCGAGGAGCTGTTCACCGGGG-3′ and GABARAP-Rv: 5′-TCACAGACCGTAGACAC-3′) was used for confirmation of the insert. The results showed that 3 founder (F0) mice were positive for the transgene. These F0 mice were backcrossed with C57BL/6J Crj 6 times to establish lines and were maintained as heterozygotes for the GFP-GABARAP transgene. One of the transgenic lines, GFP-GABARAP#901, was used for all the experiments described here. The mice were housed in pathogen-free facilities using standard animal cages with free access to standard chow. GFP-GABARAP in the mouse tissues was recognized by immunoblotting with anti-GFP, but not anti-GABARAP, antibody, while endogenous GABARAP was recognized by immunoblotting with anti-GABARAP antibody. The expression of GFP-GABARAP in mouse tissues was confirmed by immunoprecipitation with anti-GFP antibody and immunoblotting with anti-GABARAP antibody [22]. The level of expression of GFP-GABARAP in these GFP-GABARAP transgenic mice was much lower than that of endogenous GABARAP.

### Sample preparation for morphological analyses

Samples were prepared for light microscopy as described [28]. Male GFP-GABARAP mice, aged 8 weeks, were deeply anesthetized with pentobarbital (25 mg/kg i.p.) and fixed by cardiac perfusion with 4% paraformaldehyde (PA), 0.1 M phosphate buffer (pH 7.2) (PB), 4% sucrose. Immediately after perfusion-fixation, the mouse brains were excised and immersed in the same fixative at 4°C for 2 hours. Samples for cryosections were cryoprotected with 15% and 30% sucrose solutions, embedded in O.C.T. compound (Miles, Elkhart, IN) and cut into 10-µm sections with a cryostat (CM3050; Leica, Nussloch, Germany or HM560; Carl Zeiss, Jena, Germany). These cryosections were placed on silane-coated glass slides and stored at −80°C until used.

### Immunohistochemical analyses by light microscopy

Confocal immunofluorescence microscopy was performed for multiple immunostaining of sections as described [28], [29]. For single immunostaining for endogenous GABARAP, the sections were incubated with rabbit anti-GABARAP (1∶50) overnight at 4°C, followed by incubation with donkey anti goat IgG coupled with Alexa 488 (1∶400; A-21206, Life Technologies) for 1 hour at room temperature. For triple staining for ankyrin-G, MAP2 and GFP, the sections were incubated with rabbit anti-ankyrin-G (1∶50; H-215, Santa Cruz), goat anti-MAP2 (1∶100) and guinea pig anti-GFP (1∶100) overnight at 4°C, followed by incubation with a mixture of Cy5-, Cy3- and Cy2-conjugated donkey anti-rabbit, anti-goat and anti-guinea pig IgG, respectively (1∶300 each; 711-175-152, 705-165-147 and 706-225-148, respectively, Jackson Laboratory) for 1 hour at room temperature. For triple staining for ankyrin-G, calbindin and GFP, the sections were incubated with rabbit anti-ankyrin-G (1∶100; Af610, Frontier Institute), guinea pig anti-calbindin (1∶100) and goat anti-GFP (1∶100) overnight at 4°C, followed by incubation with a mixture of Cy5-, Cy3- and Cy2-conjugated donkey anti-rabbit, anti-guinea pig and anti-goat IgG, respectively (1∶300 each; 711-175-152, 706-165-148 and 705-225-003, respectively, Jackson Laboratory) for 1 hour at room temperature. For double staining for LC3 and GFP, the sections were incubated with rabbit anti-LC3 (1∶50, [28], [44] or #4108, Cell Signaling Technology) and goat anti-GFP (1∶100) overnight at 4°C, followed by incubation with biotinylated donkey anti-rabbit IgG (1∶300; BA-1100, Vector Laboratories, Burlingame, CA) for 1 hour at room temperature, and a mixture of streptavidin coupled with Alexa Fluor 594 (1∶400; S-11227, Life Technologies) and donkey anti goat IgG coupled with Alexa 488 (1∶400; A-11055, Life Technologies) for an additional 1 hour at room temperature as described [29]. For double staining for GFP and pan voltage gated sodium channel, the sections were pretreated in Liberate Antibody Binding Solution (Polysciences, Inc., Warrington, PA, USA) for 5 minutes at room temperature for antigen unmasking and then incubated with goat anti-GFP (1∶100) and mouse anti-pan voltage gated sodium channel (1∶100) antibodies overnight at 4°C. The sections were further incubated with a mixture of Cy2- and Cy3-conjugated donkey anti-goat and anti-mouse IgG, respectively (1∶300 each; 705-225-003 and 715-165-151, respectively, Jackson Laboratory) for 1 hour at room temperature. Coverslips were placed onto the stained sections with Vectashield Mounting Medium (with DAPI) (Vector) and the sections were viewed under a confocal laser-scanning microscope (FV1000: Olympus, Tokyo, Japan). As controls, sections were incubated with nonimmunized goat, guinea pig, rabbit or mouse serum diluted to 1∶100, followed by the respective secondary antibodies, or with the secondary antibodies without incubation with the primary antibodies.

### Preparation and immunostaining of cultured neurons

Cortical neurons were dissected and dissociated from embryonic day 16 mouse embryos. Dissociated cortical neurons were plated on poly-L-ornithine (Sigma-Aldrich) coated glass coverslips at a density of 25000 cells/cm^2^. Two hours later, the medium was changed from normal medium [5% FBS, 2 mM L-glutamine (Nacalai Tesque) in DMEM (Nacalai Tesque)] to maintaining medium [2% B27 (Invitrogen), 2 mM L-glutamine (Nacalai Tesque) in Neurobasal medium (Invitrogen)]. Two days later, 10 µM cytosine arabinoside (Nacalai Tesque) was added to inhibit non-neuronal growth. Seven days after plating, neurons cultured on coverslips were fixed using 4% PA buffered with 0.1 M PB (pH 7.2) and incubated overnight at 4°C with a mixture of guinea pig anti-GFP (1∶100), goat anti-MAP2 (1∶100), rabbit anti-Ankyrin-G (1∶100; Af610, Frontier Institute), and mouse anti-tau1 (1∶100) primary antibodies. The coverslips were further incubated for 1 hour at room temperature with a mixture of the appropriate secondary antibodies: Alexa405-, FITC-, Cy5-, and Cy3-conjugated donkey anti-goat, anti-guinea pig, anti-mouse and anti-rabbit IgG (1∶300; Jackson Laboratory), respectively. The coverslips were mounted onto slide glasses, coverslipped with Vectashield Mounting Medium (without DAPI) (Vector) and viewed under a confocal laser-scanning microscope (FV1000: Olympus), as above. As controls, sections were incubated with nonimmunized goat, guinea pig, rabbit and mouse sera diluted to 1∶100, followed by the respective secondary antibodies.

### Measurement of fluorescence intensity

Using TIFF images of hippocampal and cerebellar tissues triple stained for GFP, LC3 and DAPI, areas of interest, such as somata, dendrites and axon initial segments of hippocampal neurons (n = 10) and cerebellar Purkinje neurons (n = 7), were selected. The average intensity of GFP and LC3 immunoreactivity in the selected areas was measured by quantifying the averages of the green and red channels, respectively, using the Histogram tool of the Adobe Photoshop software. Intensities were normalized to those of somata.

### Statistical Analysis

For Figure 1, GFP-GABARAP dots under fed and starvation conditions were counted using an ImageJ program (http://rsbweb.nih.gov/ij/) with a TopHat plugin (http://rsb.info.nih.gov/ij/plugins/lipschitz/) on a Macintosh computer (MacBook 4.1, MacOS 10.6.8), and the numbers of dots per unit area were analyzed by Excel software (version X, Microsoft) with an add-in for one-way ANOVA to determine statistical significance.

For Figure 4M–P, differences between experimental and control groups were determined using two-tailed Student's t-tests. By using Kaleidagraph software (version 4.0 Mac; Synergy Software), the average fluorescence intensity of GFP and LC3 immunoreactivity was analyzed by one-way ANOVA to determine statistical significance. All pairwise multiple comparison procedures were performed with Tukey's *post hoc* test. Data are expressed as mean ± standard deviation. A p value<0.03 was considered statistically significant.

## Supporting Information

Figure S1 **Immnohistochemistry using anti-GABARAP antibodies.** Immunohistochemical analysis using antibodies against GABARAP raised against GST-fused human GABARAP (A, B) or synthetic peptides corresponding to residues 8–22 of human GABARAP (C, D). Endogenous GAPARAP (green) could not be detected immunohistochemically in hippocampal (A, C) and cerebellar tissues (B, D) (green). Nuclei were stained with DAPI (magenta). Abbreviations: so, stratum oriens; sp, stratum pyramidale; sr, stratum radiatum; m, molecular layer; p, Purkinje cell layer; g, granular cell layer. Bars indicate 30 µm.
(TIF)

Figure S2 **GFP-GABARAP colocalizes with voltage-gated sodium channel.** Immunopositive signals for GFP (green) and voltage-gated sodium channel (Nav) (red) co-localized in hippocampal pyramidal and cerebellar Purkinje cells located in the stratum radiatum (sr) and the molecular layer (m), respectively (arrowheads). Nuclei were stained with DAPI (blue). Abbreviations: so, stratum oriens; sp, stratum pyramidale; p, Purkinje cell layer; g, granular cell layer. Bars indicate 30 µm.
(TIF)
