# Supplementary figures and images for: Enrichment of GABARAP Relative to LC3 in the Axonal Initial Segments of Neurons
Source: PLoS One. 2013 May 9;8(5):e63568. doi: 10.1371/journal.pone.0063568 (PMC3650058; doi:10.1371/journal.pone.0063568)

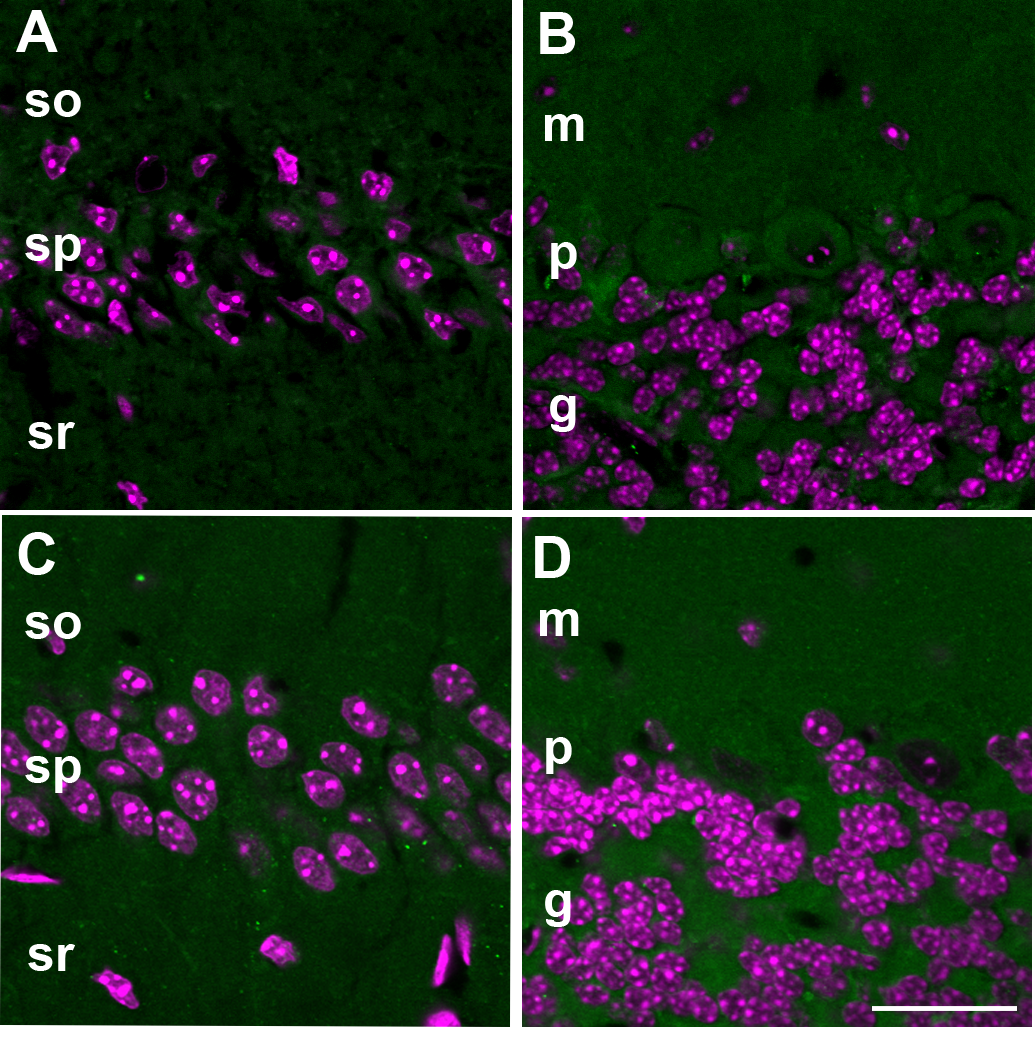

Supplement: Figure S1 — Immnohistochemistry using anti-GABARAP antibodies. Immunohistochemical analysis using antibodies against GABARAP raised against GST-fused human GABARAP (A, B) or synthetic peptides corresponding to residues 8–22 of human GABARAP (C, D). Endogenous GAPARAP (green) could not be detected immunohistochemically in hippocampal (A, C) and cerebellar tissues (B, D) (green). Nuclei were stained with DAPI (magenta). Abbreviations: so, stratum oriens; sp, stratum pyramidale; sr, stratum radiatum; m, molecular layer; p, Purkinje cell layer; g, granular cell layer. Bars indicate 30 µm. (TIF) [file pone.0063568.s001.tif]

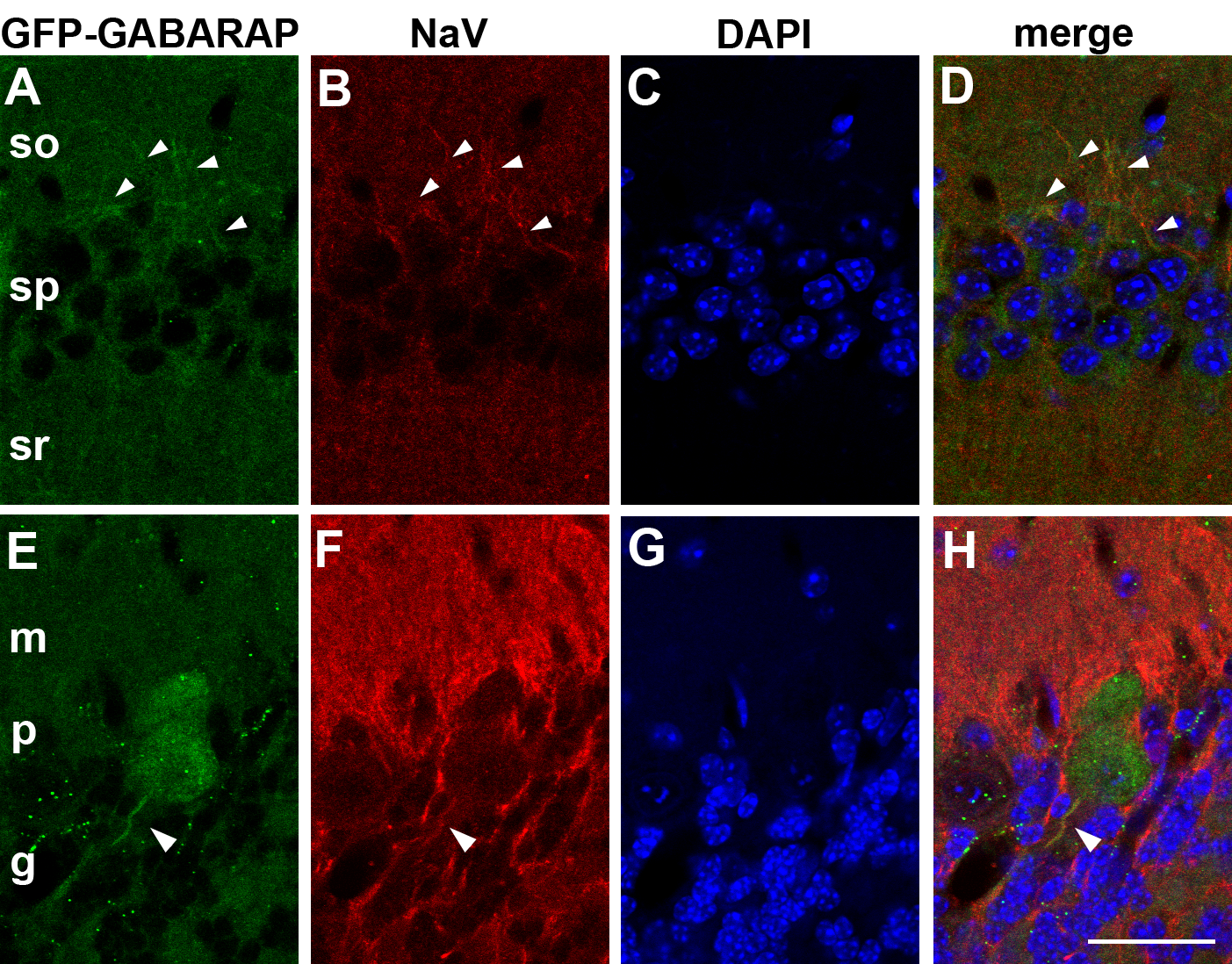

Supplement: Figure S2 — GFP-GABARAP colocalizes with voltage-gated sodium channel. Immunopositive signals for GFP (green) and voltage-gated sodium channel (Nav) (red) co-localized in hippocampal pyramidal and cerebellar Purkinje cells located in the stratum radiatum (sr) and the molecular layer (m), respectively (arrowheads). Nuclei were stained with DAPI (blue). Abbreviations: so, stratum oriens; sp, stratum pyramidale; p, Purkinje cell layer; g, granular cell layer. Bars indicate 30 µm. (TIF) [file pone.0063568.s002.tif]
